# What to Measure? Development of a Core Outcome Set to Assess Remote Technologies for Cochlear Implant Users

**DOI:** 10.3390/jcm14217697

**Published:** 2025-10-30

**Authors:** Catherine Sucher, David Allen, Emma Laird, Isabelle Boisvert, Melanie Ferguson

**Affiliations:** 1 School of Medicine, University of Western Australia, Crawley, WA 6009, Australia; 2 Curtin School of Allied Health, Curtin University, Bentley, WA 6102, Australia; 3 Incept Labs, Sydney, NSW 2009, Australia; 4 School of Health and Rehabilitation Sciences, The University of Queensland, Brisbane, QLD 4072, Australia; 5 Department of Community and Clinical Health, LaTrobe University, Bundoora, VIC 3086, Australia; 6 Sydney School of Health Sciences, University of Sydney, Sydney, NSW 2006, Australia; 7 Curtin Enable Institute, Curtin University, Bentley, WA 6102, Australia

**Keywords:** cochlear implants, adults, outcomes, core outcome set, health services, telehealth

## Abstract

**Background/Objectives:** Uptake of remote cochlear implant (CI) services is feasible in clinical studies, but implementation into regular clinical practice is limited. Effective implementation requires demonstration of at least equivalent outcomes to in-person care. Use of outcome measures (e.g., specific tools such as speech tests or surveys) that are relevant and sensitive to both modes of service facilitates evidence-based provision of CI services. Following our previous study, which developed a core outcome domain set (CODS) (i.e., a set of CI outcome areas important to measure), this study aimed to (1) review current awareness and use of outcome measures implemented clinically, in-person, or remotely; and (2) provide recommendations for a pragmatic core outcome set (COS) to assess remote technologies for CI users. **Methods:** Expert Australian/New Zealand clinical CI professionals (*n* = 20) completed an online survey regarding use of, and familiarity with, pre-identified outcome measures mapping to the previously identified CODS. Respondents rated the outcomes’ usefulness, ease of use, trustworthiness, and recommendation for future use. Stakeholder workshops (clinician, *n* = 3, CI users *n* = 4) finalised recommendations. **Results:** Four of the six most regularly used and familiar measures were speech perception tests: BKB-A sentences, CNC words, CUNY sentences, and AB words. The long- and short-form Speech, Spatial, and Qualities of Hearing Scales (SSQ/SSQ-12) were the top-ranked patient-reported outcome measures (PROMs). These outcome measures were also perceived as the most trustworthy, easy to use, and likely to be used if recommended. **Conclusions:** A pragmatic COS, relevant to both remote and in-person delivery of CI services, including recommendations for measurement of service, clinician-measured and patient-reported outcomes, and how these might be developed in future, is recommended.

## 1. Introduction

Opportunities to access cochlear implant (CI) care via remote technologies, rather than the traditional method of accessing care through in-person appointments at specialised clinics, have rapidly expanded in recent years [1,2,3,4,5,6]. Various studies have shown that both synchronous and asynchronous options for remote CI services, such as intraoperative CI telemetry, implant programming, electrode-specific measures, and post-operative assessment of speech recognition, management, and review, are possible and feasible [5,6,7,8,9]. The use of telehealth for CI service provision has the potential to significantly improve the efficiency, effectiveness, and equity of care for CI users, in a personalised manner. However, it is vital that its implementation is well-considered [1,10,11].

Common barriers to integration of remote care into hearing services for either CIs or hearing aids include a lack of adequate funding frameworks; poor integration with current clinical practices; mistrust of the accuracy and quality of the remote care service; measures and outcomes; and audiologists’ confidence in their clients’ ability to utilise the remote technology [1,2,12,13,14]. Nevertheless, most studies show that use of a hybrid system in which both remote and in-person care is provided is the preferred method of service delivery, both by clients and audiologists [9,13,14,15].

In order to implement remote care effectively into clinical practice, it is essential to demonstrate that remote service provision provides equivalent, if not superior, care compared to the current standard of in-person clinical care [16]. This is necessary for regulatory purposes, as well as to ensure that CI service providers and users have sufficient trust and confidence in the outcomes of the remote service to consider using it.

Within audiology, a vast number of outcome measures exist for measuring the effectiveness of CIs and hearing aids [17,18,19]. Danermark et al. [20] suggested a concise set of outcome measures for assessment of hearing in general, and Allen et al. [21] identified a core outcome domain set (i.e., a set of patient outcome areas that should be assessed, but not specific tools for assessment) for hearing rehabilitation, primarily for hearing aids. These sets of measures and domains, however, do not address some of the specific auditory issues associated with severe–profound hearing loss or the technical issues associated with the use of CIs. More specific to CIs, Andries et al. [22] recommended a CI-specific outcome assessment protocol for adult CI users, which an expert group of CI professionals selected based on the WHO international classification of Functioning, Disability, and Health (ICF) framework. The ability to assess outcomes for CIs, such as speech perception, is particularly problematic in remote care, given the difficulties determining presentation levels and establishing standardised test environments (e.g., a sound-treated booth), compared to in-person clinical measures. Currently, there is no set of measures for CI-specific outcomes when also used in combination with remote technology.

The use of relevant and sensitive outcome measures to evaluate CI services delivered via remote technologies is vital to facilitate the provision of evidence-based healthcare services, allowing stakeholders to make informed decisions about how to best care for their patients. The current approach to audiological outcome measures is essentially non-standardised [23], both for in-person and remote services, making it difficult to compare and integrate results across different studies and services, for example, in systematic reviews with meta-analyses.

To address these issues, over the last decade, there has been an increase in the development and use of core outcome sets (COSs) [24]. A COS is an agreed, standardised set of outcomes that should be measured and reported as a minimum dataset for a specific condition [25], ideally with input from end-users, including patients, clinicians, industry, and other key stakeholders. Outcome measures (specific measurement tools) are identified as part of pre-specified outcome domains (i.e., CI outcome areas which have been deemed important to measure by CI users and CI professionals—i.e., what should be measured, but not specific measurement tools). A core outcome domain set (CODS) has been defined for hearing aids with separate, and significant, input from both patient and hearing care professional stakeholder groups [21] based on best practice guidelines [26]; however, there is nothing similar for CIs. Traditionally, CI outcomes have focused on the domains of speech perception and CI uptake, although there is growing evidence that patient-reported outcome measures (PROMs) offer a more functional real-world outcome, tapping into different mechanisms of benefit to speech perception outcomes [27,28]. More recently, there has been a growing number of PROMs specific to cochlear implants [29,30], although the extent of how they are used in clinical practice is unclear.

With the increase in use of remote technologies, there is a need to consider the meaningful domains that are specific to these technologies, which may also be relevant to in-person services. For example, empowerment has recently emerged as a feature of remote technologies [31,32], but it likely also applies to in-person services. Furthermore, there are other considerations specific to the service delivery of remote technologies that are often identified as benefits to both patients and services, such as reduced time, convenience, and costs [1,2,12,14].

**Figure 1:**
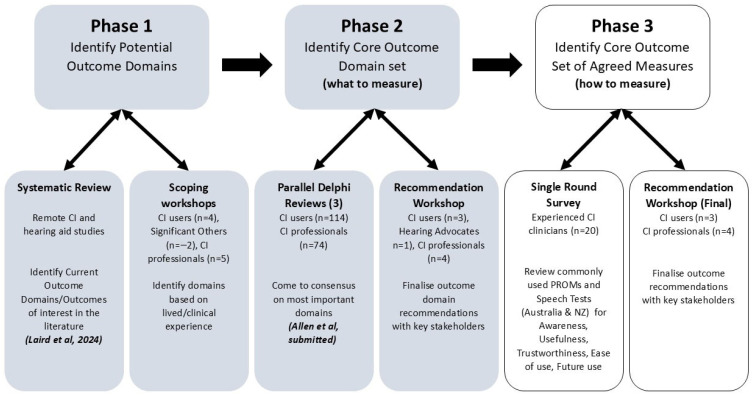
The three phases of the study [28,33].

An essential tenet of this research is to be able to demonstrate the equivalence of remote care services, either as a stand-alone or hybrid model of care, to the ‘gold-standard’ in-person clinical model of care. Thus, the overall objective of this research was to develop a COS to evaluate remote technologies delivered within CI services to maximise the potential benefits of this model of care. Feedback was sought from relevant parties (e.g., CI users and their families’ service providers, including management and clinicians, CI manufacturers, and CI advocacy groups) to ensure a broad range of perspectives were considered. Outcome domains encompassing outcome measures (measurement tools) specific to the delivery of remote technologies, in terms of both (i) patient outcomes (i.e., benefits of remote technologies for CI patients) and (ii) service delivery, were included, ensuring the outcomes can be easily integrated into clinical care. This paper reports on the final phase of a broader three-phase study (see Figure 1), which used the COS development roadmap described by Hall et al. (2015) [26] as the theoretical underpinning. The current study followed Stage 2 (identify and agree on outcome measures) of the Hall et al. (2015) [26] roadmap, where outcome measures were systematically appraised based on COSMIN principles. The study was registered on the COMET (Core Outcome Measures in effectiveness Trials) website: https://www.comet-initiative.org/Studies/Details/2586 (accessed on 1 October 2022).

Phase one of the study included a systematic review of outcome measures identified in studies documenting the use of remote services for the provision of CI and hearing-aid care [28]. A total of 250 different outcome measures were identified, with CI studies revealing significantly more outcomes in the ear and labyrinth domains, compared to hearing-aid studies (43% vs. 10%), and hearing-aid studies revealing significantly more outcomes in the cognitive (28% vs. 5%) and emotional (35% vs. 10%) domains than CI studies.

Phase two involved a combination of stakeholder workshops with CI users and their significant others, CI professionals, and hearing advocates, followed by a series of three parallel e-Delphi reviews conducted separately for 74 CI professionals and 114 CI users across Australia, the UK, and the USA. This utilised a methodology described by Allen et al. [33] outlining the development of a CODS for adult CI outcome domains. This phase aimed to identify, by consensus, the most important outcome domains based on stakeholder input [33]. The Delphi review assessed 58 domains across three supradomains: Service, Clinical (assessment-based), and Patient (self-report). The top three domains, in which a consensus of ≥80% was achieved within each supradomain for both groups (i.e., CI users and CI professionals), are shown in Table 1. Agreement was good for the Service supradomain; however, the consensus was poorer for the Clinical supra-domain, and there was no between-group agreement for the Patient supra-domain. Many domains ranked highly by CI users were ranked far less important by professionals.

The aim of the final phase of the study reported in this paper, Phase three, was to identify a COS to evaluate remote technologies delivered within CI services based on the previously defined CODS. Due to the substantial mismatch in outcome domains for both the Clinical and Patient supra-domains between CI users and CI professionals noted in Phase two, Allen et al. [33] recommended inclusion of domains ranked most highly by CI professionals for the Clinical supra-domain, and by CI users for the Patient supra-domain in an interim, pragmatic COS. This would facilitate an easy transition into a robust, pragmatic, and clinically acceptable COS utilising clinical measures used regularly and trusted by CI programmes across Australia and New Zealand.

## 2. Materials and Methods

Outcome measures that mapped onto the CODS were selected from those identified in Phase one, and outcome measures identified by the research team as commonly used in clinical care in Australia and New Zealand were included in Phase three of the study. The list of identified outcome measures was appraised based on the Hall et al. (2015) [26] roadmap. Content validity and developmental methodology were used to identify which outcome measures were included in Phase three. The final list of included outcome measures, consisting of 43 patient-reported outcome measures (PROMs) and 10 speech perception outcome measures (speech perception tests) (see Supplementary Tables S1 and S2), was presented to experienced CI clinicians from Australia and New Zealand in a single-round online survey. Each clinician was asked to rate the outcome measures for their use of the measure and, if used, the outcome measures’ usefulness, trustworthiness, ease of use, and likelihood of future use if it were recommended to them.

### 2.1. Single-Round Online Survey

Expert CI clinicians (*n* = 57) from large adult CI services in Australia involved in the provision of CI services in Australia and New Zealand were identified by authors CS and IB from professional contacts and invited to participate via email invitation. Clinicians were excluded if they had less than 12 months of experience in adult CI service provision or did not work with adult CI recipients. Individuals who agreed to participate completed a single-round online survey utilising Qualtrics software, Provo, UT, USA, https://www.qualtrics.com/, accessed date 1 November 2023. The online survey consisted of questions about the use of 10 speech perception tests and 43 patient-reported outcome measures (PROMs) using a 4-point categorical scale (never heard of, never used, occasionally used, regularly used). A short description was provided for each PROM listed. For example, “The Social Participation Restrictions Questionnaire (SParQ) is a hearing-specific, patient-reported outcome measure that was originally developed through consultation with adults with hearing loss, clinicians, and researchers. It has 19 items, each assessed on an 11-point scale. Responses are averaged to form two subscales: Social Behaviours and Social Perceptions”. A copy of the Example Survey is available in Supplementary Materials.

Eligibility criteria for participants were as follows: recent or current CI clinicians from a range of large CI clinics and research institutes, and identified by the research team as having extensive knowledge of current CI clinical practices and/or extensive knowledge of currently available adult-focused CI outcome measures used in Australia and New Zealand. Clinicians included experienced clinicians who participated in Phase two [33]. They were invited to participate via an email message.

When participants indicated that they had regularly, or occasionally, used a measure, they were asked to provide a rating, using a 5-point Likert scale, ranging from “strongly disagree” to “strongly agree” for each of the following statements:

1. This measure is easy to use in clinical practice (ease of use);
2. This measure gives results that are trustworthy/believable;
3. This measure gives results that are useful in clinical practice;
4. I would use this measure in clinical practice if it were recommended to me.

Respondents were also asked about other clinical outcome measures they used as part of their standard protocol, their usual approach to testing asymmetrical hearing losses, and the factors that they considered when choosing a speech test to ensure that no outcome measures were missed.

Statistical analysis was conducted in Python (v3.11.0) [34], using pandas (v2.2.1) [35], numpy (v1.23.5) [36], and scipy (v1.11.4) [37], and scikit-learn (v1.4.2) [38]. Graphics were generated using matplotlib (v3.8.4) [39]. Descriptive statistics were calculated for demographic variables and survey responses.

### 2.2. Online Final Recommendation Workshops

Two Final Recommendation workshops, approximately 90 min each in duration, were held online through Microsoft Teams videoconferencing software, WA, USA (version 1.6.00.29964) with CI users and CI professionals to finalise key recommendations for the interim pragmatic COS. The outline presentation of the study was provided, one for professionals, and a layperson version for CI users, summarising the key results from the CODS and the current study. A semi-structured interview guide (see final workshop agenda in Supplementary Materials) was developed. Questions included the following:

1. What domains should be included in future iterations of the COS?
2. (CI professionals only) Which outcome measures or subdomains should be recommended as a minimum standard?
3. How should we prioritise outcome measures within each subdomain?

Participants consisted of two groups: (1) Adult CI users and (2) CI professionals. CI professionals were required to have at least 12 months of experience providing CI services to adult CI users. Adult CI users were required to be ≥18 years of age with at least 6 months experience using a CI and sufficient self-reported English proficiency to participate in the workshop. Individuals with self-reported disability, other than hearing loss, that precluded full participation in the workshop were excluded. Potential participants were invited, via email, from the list of individuals who had participated in Phase two of the study and agreed to participate in Phase three.

## 3. Results

### 3.1. Single-Round Online Survey

Twenty CI clinicians from Australia (*n* = 18) and New Zealand (*n* = 2) participated, 17 of whom responded to both the PROM and the speech perception test familiarity survey questions. Participants’ clinical and research experience is detailed in Table 2. Home and work postcodes for Australian participants were mapped to the Index of Relative Social Advantage and Disadvantage (IRSAD) decile, with all Australian participants living in the top 30% of postcodes and working in the top 50% of postcodes, suggesting that participants skewed toward relative social advantage. For participants from New Zealand, postcodes were mapped using the New Zealand Index of Deprivation for 2023 [40], with one participant living in the top 30% of postcodes but working in an inner-city location in the bottom 30% of postcodes, and the other participant living in the bottom 30% of postcodes but working in the top 50%.

#### 3.1.1. Familiarity Ratings (Speech Perception Outcome Measures and PROMs)

A summary of familiarity ratings is shown in Table 3. Participants were most familiar with speech perception outcome measures. Four speech perception outcome measures (BKB-A, CUNY sentences, CNC words, and AB words) fell within the top five most-used outcome measures and were regularly used by 50% of participants. The DIN/DTT test, BKB-SIN, and QuickSIN were occasionally used by >50% of participants. The remaining three speech perception tests (HINT, Austin, and AzBio) had either been “never heard of” or “never been used” by 59%, 53%, and 76% of participants, respectively.

Familiarity with PROMs was lower than for speech perception outcome measures. The SSQ (90% of participants) and the SSQ-12 (75% of participants) were the most used PROMs. Participants used the SSQ-12 (70%) slightly more regularly than the SSQ (65%). The Glasgow Hearing Aid Benefit Profile (GHABP), Hearing Handicap for the Elderly (HHIE), and Abbreviated Profile for Hearing Aid Benefit (APHAB) were occasionally or regularly used by at least half the participants. No participant regularly used the short or revised version of the HHIE. The Nijmegen Cochlear Implant Questionnaire [53] (NCIQ), and the Cochlear Implant Quality of Life Questionnaire (CIQoL Profile and CIQoL-Global) [30], which have been recommended by the Adult Hearing Standards of Care; Living Guidelines [88], were not commonly used. The NCIQ was only used by 5% of participants regularly and by 45% occasionally. The CIQoL was used only occasionally by 35% (Global version, 35 items) and 20% (Profile version, 10 items) of participants.

#### 3.1.2. Ease of Use, Trustworthiness, Usefulness, and Likely Recommendation to Use Ratings

**Figure 2:**
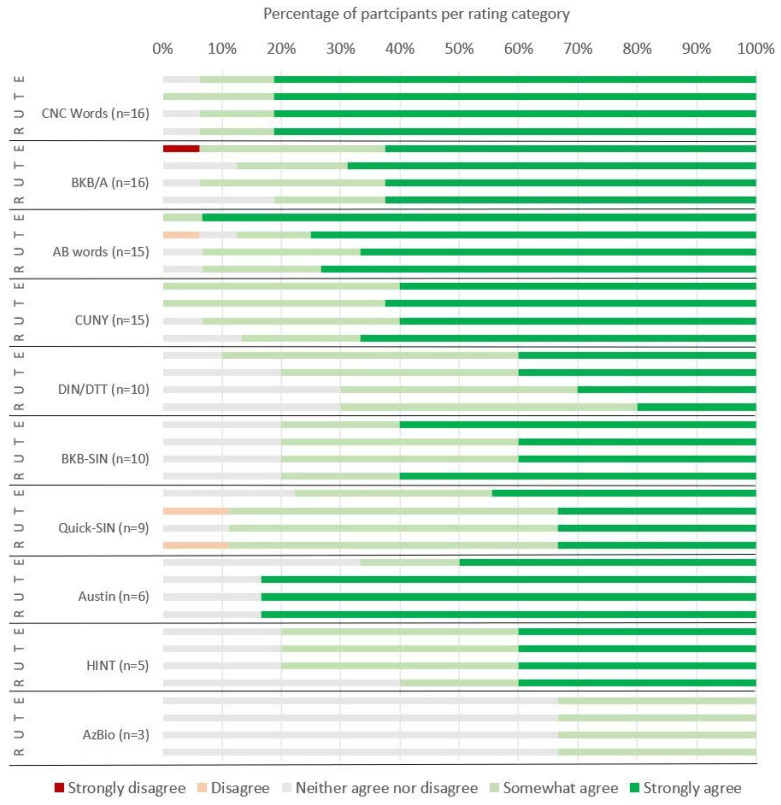
Speech perception outcome measures. Ratings for ease of use (E), trustworthiness (T), usefulness (U), and likelihood of future recommendation for use (R). Number of participants who had used each measure, either occasionally or regularly, and thus provided ratings, is shown in parentheses after the named outcome measure. For abbreviations, see Table 2.

**Figure 3:**
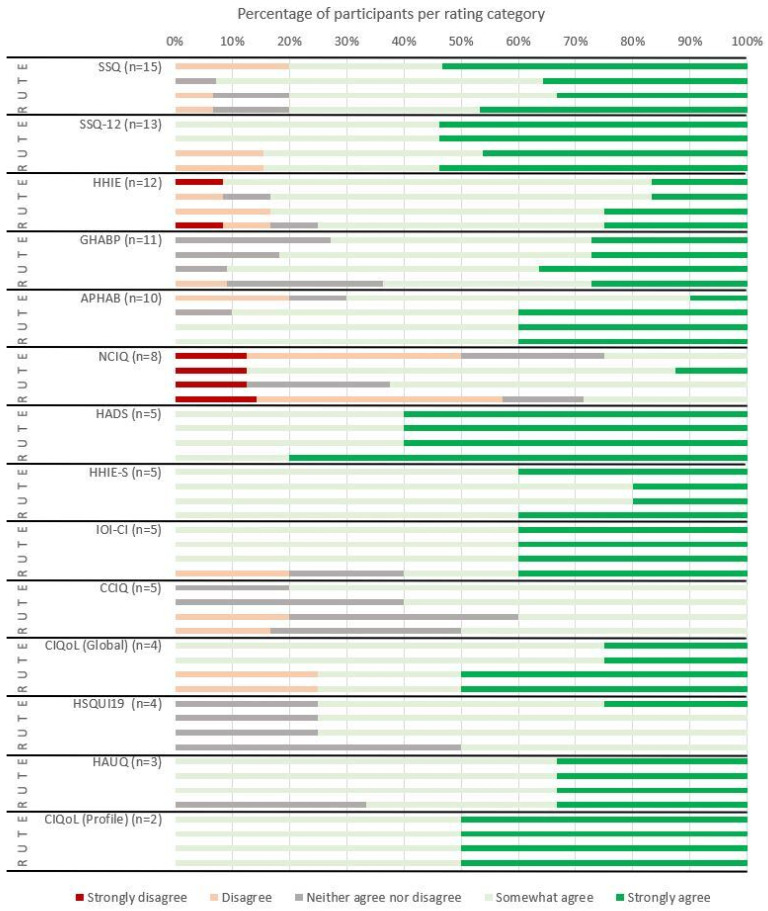
Patient-reported outcome measure (PROM) ratings for ease of use (E), trustworthiness (T), usefulness (U), and likelihood of future recommendation for use (R). Number of participants who had used each measure, either occasionally or regularly, and thus provided ratings, is shown in parentheses after the named outcome measure.

Ratings were provided for ten speech perception outcome measures (Figure 2), and 21 PROMs (Figure 3), which had been used by ≥3 participants. The CIQoL Profile ratings were also included, although they had only been used by two participants. Outcome measures with which participants were more familiar were, in general, considered easier to use (τ_B_ = 0.383, *p* < 0.001), more trustworthy (τ_B_ = 0.323, *p* < 0.001), and as providing more useful results (τ_B_ = 0.300, *p* < 0.001). Participants also reported that they would be more likely to use them in practice if they were recommended (τ_B_ = 0.406, *p* < 0.001) (Figure 3). Ratings were generally high, with very few respondents disagreeing with any of the statements.

The correlation between the ratings of ease of use, trustworthiness, clinical usefulness, and willingness to use outcome measures was assessed using univariate and bivariate kernel density plots, and correlations between all rating scales were high (see Supplementary Figure S1).

#### 3.1.3. Free Text Responses

Participants suggested several additional PROMS not listed in the survey (Supplementary Table S2), including the Australian Quality of Life Scale (AQoL; *n* = 4), the Strengths and Difficulties Questionnaire (*n* = 3), and the Listening Effort Questionnaire (LEQ-CI; *n* = 3). The most recommended physiological test was Neural Response/Auditory Response Telemetry (*n* = 3). The Ling Sounds speech sounds identification test was also suggested (*n* = 3).

The most important factors to consider when choosing a speech perception test (see Supplementary Table S3) were as follows: tests available in the primary language (*n* = 11), accent (*n* = 7) of the CI user, cognitive appropriateness (*n* = 4), speed of delivery (*n* = 2), and measure length (*n* = 2) (see Supplementary Table S3).

### 3.2. Final Recommendation Workshops

Transcriptions of the final workshops were reviewed and summarised by authors CS and MF, then reviewed by all other authors. Key findings from the workshops are shown in Table 4.

Clinicians felt that future outcome measures should include domains such as cognition, listening effort, listening fatigue, empowerment, social connectedness, relationships, and fatigue. There was a general consensus between both groups that more holistic measures of CI outcomes are needed to provide a more comprehensive understanding of the communication difficulties of CI users and their real-life impact. A combination of different types of outcome measures and understanding their interactions was considered crucial for advancing the field and improving clinical outcomes. However, with the emergence of newly developed outcome measures, interpretation of results may be challenging due to a lack of clinician familiarity. Thus, when considering the introduction of new outcome measures, it is vital to consider training as part of implementation, to raise awareness, familiarity, and ensure trust in the data obtained with the measure.

CI users felt that interaction with the clinician in some aspect of the remote service was important to feel engaged in the process. They recommended that remote services be well-considered and designed to ensure a seamless process for all aspects of the service, from enrolment, validation of enrolment, and login to completion of the remote checks, appointments, payment, etc. Whilst benefit was seen in the ability to adjust CI settings remotely, CI users reported it was essential that there was a built-in fail-safe or reboot option at the CI user’s end if the service failed midway for some reason.

## 4. Discussion

Whilst remote care has been shown to be a feasible option for CI service provision, the uptake and sustained use of such services have remained low. In order to compare remote and in-person services effectively, as well as address the concerns about accessibility, usability of services, and accuracy of results, it is essential that the same set of outcome measures that are sensitive and meaningful to both CI users and CI professionals are compared across clinics, modes of service provision, and clinical trials/studies.

While the COS recommended by Andries et al. [22] included CI-specific outcome measures, the measures and domains selected for inclusion were not selected with the input of CI users but rather by a core group of CI experts. While well-known, commonly used instruments and assessment methods were identified, several of the PROMs selected were not designed according to the current recommended best practice, e.g., using consumer input, considering the risks of bias, or following evidence-based criteria for good psychometric measurement properties [89], nor were they CI-specific. Finally, the outcome measures recommended did not specifically consider implementation within a remote care service and the issues associated with this. The COS [22] included a large number of outcome measures: PROMs (Work Rehabilitation Questionnaire [WORQ; 59 items], Abbreviated Profile of Hearing Aid Benefit [APHAB; 24 items], Audio Processor Satisfaction Questionnaire [APSQ; 15 items], Speech Spatial and Qualities of Hearing Questionnaire [SSQ-12; 12 items], Hearing Implant Sound Quality Index 19 [HISQUI19; 19 items]), and included audiometric outcome measures: Aided Pure tone audiometry, Speech perception [Monosyllabic words in quiet, Sentences in noise], and Sound localisation.

Several of the domains identified as most important by CI users and CI clinicians in the earlier stages of our study [33] were not included in the domains in the above-mentioned COS; of particular note are device integrity and status, device use, and hearing-related quality of life. Understandably, remote service domains of reliability, accessibility, and ease of use were not included. In Phase two, a complete lack of consensus between CI users and CI professionals was observed in the most important domains for self-report “Patient” outcome measures, and there was only a limited consensus for objective “Clinical” outcome measures. Thus, outcomes recommended in the COS by Andreis et al.’s [22] may not be important to CI users, given the lack of CI user input. Further, our final recommendation workshop revealed a consensus between CI users and CI clinicians for a minimalist approach to the number of core outcome measures. One must consider the potential for overburdening CI users and clinicians with multiple outcome measures, particularly PROMs with large numbers of items, some of which ask similar questions. A large number of time-consuming outcome measures may result in poor completion compliance and inefficient or limited clinical use of completed outcome measures. Outcome measures completed or received immediately prior to, or during, an appointment, particularly PROMs, may be difficult and time-consuming for the clinician to analyse appropriately during the appointment.

Although the original aim of this study was to develop a COS for CI users utilising remote technology in Australia and New Zealand, this has proved difficult for several reasons:

1. Lack of consensus between CI users and CI professionals on the most important domains for the patient supra-domain [33] means that the implementation of a concise COS is problematic if one is to measure the most important domains within each supra-domain.
2. Lack of well-designed and/or well-validated outcome measures for some of the domains rated as most important to assess. Rigorous development and assessment of novel outcome measures is, therefore, required.
3. Current clinical practice trends in Australia and New Zealand, observed in our online survey of CI clinicians, indicate that CI services rely predominantly on a relatively small pool of specific speech perception outcome measures as the primary measure of CI outcomes. Clinicians are far less familiar with most PROMs that align with the CODS.
4. Several of the outcome measures identified and explored in the outcomes survey have proprietary test materials, technical requirements, and licencing costs associated with them.

These issues present several problems when considering the widespread implementation of a COS for remote technology into well-established CI clinics. Large CI clinics often perform retrospective analysis of CI outcomes over time as an important indicator of the success of both individual CI users and CI clinics as a whole [90]. Thus, implementation of a brand-new set of outcome measures must consider the impact on the ability to compare outcomes over time. It may be necessary to align the outcomes of new outcome measures with pre-existing outcome measures for a period of time in order to retain the ability to compare outcomes over time. Significant support, resources, and training regarding outcome measures that are new to the field, or simply new to the clinic, will be required.

Clinicians must have confidence and trust in the recommended outcome measures, both in the methods of data collection and interpretation of results, as evidenced by the high levels of correlation between the ease of use, trustworthiness, usefulness, and recommendation ratings provided by participants. Familiarity with an outcome measure engenders an understanding of effective methods of use and interpretation, and, in turn, outcome measures that are inherently easier to apply and interpret are more likely to become part of the existing clinical practice [91,92,93]. Outcome measures that provide trustworthy results are also more likely to be considered useful in clinical practice. Understanding which of these four factors of clinician experience, if any, are primarily responsible for positive clinician experience and uptake is essential to support implantation efforts. This is particularly salient, given our findings in relation to some more commonly used surveys, such as the CIQoL and the NCIQ, both of which were recommended outcome measures in the recently drafted Adult Hearing Standards of Care; Living Guidelines [88]. Our study revealed poor ratings for ease of use, likelihood to use if recommended, and, to a lesser extent, usefulness for the NCIQ, which would indicate that it is unlikely that this measure would be readily adopted into CI clinical practice in Australia or New Zealand. Furthermore, the NCIQ contains 60 items and so fails to meet the recommendations of our CI users about shorter, more concise outcome measures. The CIQoL, whilst receiving relatively good ratings for all four categories, had only been used by a maximum of four clinicians; thus, training for its implementation is required.

In light of these considerations, it appears most appropriate to recommend a pragmatic, interim COS for remote technologies for CI users in order to facilitate uptake into current clinical practice, with the recognition that CI outcomes are constantly evolving [88], and as such, so are the important outcome domains, and outcome measures with which to assess them are also evolving. Furthermore, it was decided to limit outcome measures to those commonly used in English-speaking countries in the first instance, as Australia and New Zealand were the focus of this study, to further facilitate compliance with the use of the COS, as commonly used outcome measures differ substantially across countries. There was a strong focus in the workshops on the need to ensure that implementation of any new set of outcome measures did not overburden either CI users or CI clinicians. Whilst there was a push to utilise more meaningful, “real-life” outcome measures, this was not to be at the expense of additional time and effort for key stakeholders. In fact, the preference was for a reduction in time allocated towards the assessment of outcomes. Similar findings have been noted in other allied health fields [94]. However, a reduction in the length of speech test lists, or the number of questions in surveys, should not be at the expense of a reduction in their psychometric properties, such as test–retest reliability and validity. Any recommended outcome measures must have and retain good psychometric properties to ensure their usefulness.

### 4.1. Recommended Interim, Pragmatic COS

#### 4.1.1. Service Outcomes

A single Likert item was assigned for reliability, usability, and acceptability, with an option for free text, as recommended in stakeholder workshops.

The wording of the scale needs to be further defined, but a regularly mentioned example was a five-response option based on agreement within stakeholder workshops (e.g., the remote technology was reliable: strongly agree to strongly disagree).

#### 4.1.2. Clinically Measured Outcomes

The CNC (or similar CVC) and optional digit triplet testing (DTT), testing device integrity/system check, device use, and adverse events.

Recommended outcome measures are as follows:

An adaptive speech test that is presented in noise but could also be completed in quiet, depending on the CI user’s speech perception ability. Of the four most regularly used tests identified by CI professionals (BKB-A, CUNY, CNC, and AB), the CNC test was included, as this is a current requirement for CI candidacy determination. It is, however, noted that work is required to ensure appropriate integration of a “remote” version of the CNC word lists into the remote clinical workflow.

The DTT is a speech-in-noise test that is often delivered remotely, thus having the appropriate underpinning architecture for delivery via remote technology systems. Other advantages of this test include that it is often delivered adaptively, the digitized material is easily translatable into other languages with easily understandable stimuli, it can be delivered without the need for calibration equipment, and it has been widely used and validated across the world.

Device integrity and status, device use, and adverse events were the other three most highly rated clinical tests, in addition to speech perception testing. Both groups felt it was vital to ensure that both internal and external components of the CI were functioning appropriately (device integrity) to ensure that any outcome measures added to these measures are not impacted by device malfunction. Device use, via datalogging, has been included in the COS to ensure that limited CI outcomes are not the result of limited CI use. We acknowledge, however, that there are known discrepancies between reported use and logged data, and that this discrepancy could be due to either technological errors or the user’s decision not to report limited use.

Any potential worry about device usage should be discussed in a supportive and caring manner with the CI user.

Outcome measures excluded include the following:

The BKB-A test (quiet and fixed S:N) was excluded because of the fixed-level presentation of sentences and the fact that it was originally developed for a low (kindergarten age) literacy level. It is somewhat child-like, resulting in ceiling effects, and its typical presentation mode is quiet. Significant ceiling effects were reported by Gifford et al. [95] when assessing adult CI users with the HINT, which consists of selected BKB/A sentences, presented in quiet and at fixed levels of noise.

The CUNY sentence test was excluded due to ceiling effects relating to the high level of predictability of sentences seen amongst post-lingually deafened adult CI users [96] and the relatively high language knowledge/literacy level required, in addition to the potential influence of auditory memory on outcomes.

The AB word test [43] was excluded due to the limited number, 15, of short (10-word) lists available, in addition to the widespread use of this test material throughout hearing clinics in Australia and the lack of evidence-based empirical data on their use, which could lead to practice effects [97]. Furthermore, the Australian accent version of the test materials is no longer available for purchase from the National Acoustic Laboratories.

BKB-SIN and Quick SIN were excluded because of the lack of accessibility to recordings in an Australian accent and the inclusion of words not routinely used in Australian English, as per final workshop recommendations from both CI users and CI clinicians.

Speech sound identification/discrimination assessment (e.g., the LING test), whilst rated highly by CI users, was perceived by CI professionals as a more diagnostic measure to indicate specific hearing difficulties, rather than an overall measure of CI outcome, and thus was not included in the current interim COS.

#### 4.1.3. Patient-Reported Outcome Measures (PROMs)

Given the discrepancy in domain importance between CI users and CI professionals in this supra-domain, preference was given to CI users based on feedback provided in Phase two, in which it was suggested that CI users’ everyday life experiences should be prioritised.

Recommended outcome measures are as follows:

The SSQ or short-form SSQ-12 is an interim PROM because it is the most regularly used, easy to use, trustworthy, and most likely to be used if recommended in the interim, whilst other PROMs gain clinical acceptance and implementation. CI professionals also felt that, in the context of remote services, given the potential impacts associated with variability in the home test environment at each test point, there was a necessity to have a reported measure of hearing ability to confirm the behavioural test measure. The SSQ-12 has been recommended in the ANZ adaptation of the Adult Standards of Hearing Care; Living Guidelines ANZ adaptation [98]. However, it should be noted that it does not address the most important domains of hearing-related quality of life, satisfaction, and wellbeing. Thus, although it is commonly used, this alone is not an appropriate criterion for its long-term inclusion in future COSs.

Hearing-related quality of life, satisfaction, and wellbeing are outcome domains for consideration. Based on these domains, the CIQoL (Cochlear Implant Quality of Life) [30] would be a suitable PROM, augmented by a satisfaction measure. The CIQoL is a well-validated outcome measure developed with stakeholder input, using modern psychometric Item Response Theory analysis. The CIQoL Profile (35 items) has sub-domains: hearing, communication, social relationships, emotional wellbeing, independence and daily life, device satisfaction and use, cognitive and mental engagement, and perception of self and identity. It is not a unidimensional measure (i.e., quality of life), but the authors suggest the broader sub-domains reflect quality of life. Alternatively, the Living with Cochlear Implants (LivCI) [99], a recently developed CI-specific, 22-item PROM, which includes four sub-domains addressing psychosocial and wellbeing, participation (i.e., HRQoL), aesthetics and visibility (a primary driver of satisfaction), and stigma, could be considered. Like the CIQoL, the LivCI has been developed according to COSMIN best practice principles, including extensive stakeholder (e.g., CI professionals and CI users), content evaluation, and contemporary Rasch analysis to ensure high-quality items that are independent, alongside Classical Test Theory analyses. Either or both of these outcome measures, which address several of the most important “Patient-reported” outcome domains identified as part of our CODs, should be included alongside the SSQ-12 following appropriate training and implementation into the clinical setting, and could be potential candidates to replace the SSQ in the future. Furthermore, both PROMs are recommended for use in the ANZ adaptation of the Adult Standards of Hearing Care; Living Guidelines ANZ adaptation [98].

In the absence of an appropriate PROM for CI user satisfaction with devices, it was suggested that, as for Service outcome measures, a Likert single-item measure could be used in the interim.

Of the numerous PROMs assessed in this study, many were not familiar to or trusted by experienced CI clinicians. Furthermore, most (see Supplementary Table S1) were not designed specifically for use with people who were users, or potential users, of cochlear implants. Some, although designed specifically for CI users/candidates, had not been well-designed according to best principles, such as inclusion of CI user input during item selection, unidimensionality of items, or appropriate scale sensitivity. Finally, an important consideration brought up in recommendation workshops was the need to ensure that CI users and CI clinicians, as stated in the final workshop, were not overburdened with surveys that included excessively large numbers of items, asking similar questions several different ways, and thus making them prone to poor or invalid completion and difficult to analyse and utilise effectively within a clinical setting. In the next version, a broader stakeholder involvement in a final version is recommended.

#### 4.1.4. Future Considerations for Validation, Revision, and Expansion of the COS

Other domains that are emerging as important for remote technologies within audiology [21], but not widely considered in the CI field, such as empowerment, listening effort, and auditory fatigue, should also be considered for a future COS. There are a number of well-developed CI- or hearing-specific PROMs which address such domains. Additionally, an assessment of digital literacy, whilst not an outcome measure per se, prior to CI users using remote technologies should also be considered.

A noted limitation of our study is the limited generalizability of the current version of the COS, developed for the Australian/New Zealand context, to international markets. Given differences in language requirements of test materials, in addition to differing candidacy for CI, at present, we believe it is particularly difficult to create a generalised COS for an international market. This is particularly evident with the recent publication of ANZ-specific guidelines for Adult CI management [98]. To overcome this, adaptation and validation of COS-recommended outcome measures into multiple languages are required. This said, the results of this study can inform CI COS development internationally, for example, by highlighting the importance of standardising outcome measures for both in-person and remote assessments, the preference for a reduced length of assessments, and the likelihood of different outcome prioritisation between CI users and professionals.

It is noted that, despite the increasing availability of COSs across the medical and allied health landscape, their use is often limited. A number of barriers to the uptake of COSs have been reported, including a lack of awareness of the COS, poor integration of outcome tools into clinical workflows, and limited incentive for adherence [100,101]. Education is therefore a vital component of uptake of the COS across CI centres, as is the use of early adopters of the COS to “champion” its use within the clinical setting. Development of templates or tools that integrate COS measures, particularly electronically, will also greatly enhance its use [101]. Finally, it is recommended that the COS measures be utilised for voluntary audit of CI outcomes across clinics, allowing clinics to benchmark outcomes against each other, and incentivising the use of the COS.

It is strongly recommended that ongoing monitoring of the clinical practices and opinions of CI clinicians is carried out every five years to ensure the advancement of clinical practice. This may be possible through professional groups, such as Audiology Australia. Adoption of future COS iterations into national guidelines, such as the recently published ANZ National Living Guidelines, will also greatly enhance awareness of the COS. A part of this process would be to update the interim COS recommended here over time, as well as to guide the development of policy and ongoing implementation, training, and de-implementation within clinical practice.

## 5. Conclusions

Development of a core outcome set (COS) to assess remote technologies used by CI users is vital, given the increase in the use of remote technologies for CI care. It is important that such a COS is relevant across both remote and in-clinic services to enable comparison and seamless integration of the two modes of service. It must also incorporate meaningful, useful outcome measures for CI users, their families, and CI clinicians alike, using well-designed, trusted outcome measures that can be incorporated into clinical practice without unnecessarily overburdening staff, CI users, or their families. We present a pragmatic, interim COS for use in hybrid clinical practice, noting that ongoing monitoring of meaningful future outcomes and clinical practices may result in adaptations to the recommended COS in the future.

## Figures and Tables

**Table 1:** Core outcome domains (CI outcomes areas, or “what” to measure) identified in Phase two [33]. * Equal rating of second for both domains by CI users. HL: hearing loss; CI: cochlear implant.

	Supra-Domain					
	Service		Clinical		Patient	
Domain Priority	CI Users	CI Professionals	CI Users	CI Professionals	CI Users	CI Professionals
**First**	**Reliability** of remote technology	**Usability** of remote technology	**Speech recognition** **in noise**	**Device** **integrity and status**	**Participation restriction** due to HL	**Expectations of hearing health outcomes**
**Second**	**Usability** of the remote technology	**Accessibility** of the remote service (for CI user)	**Speech recognition** **in quiet**	**Speech discrimination**	**Hearing Related Quality of Life** AND * Satisfaction with CI	**Motivation and Readiness to Act** on hearing difficulties
**Third**	**Accessibility** of the remote service (for CI user)	**Reliability** of remote technology	**Speech discrimination**	**Device Use**	**Mental Health and Wellbeing**	**Acceptability and Tolerability** of the CI (for CI user)

**Table 2:** Clinical and research experience of CI clinicians who participated in the survey.

	Number of Participants (%)	Median (Years)	Range (Years)
Duration of Clinical Audiology Experience	20 (100%)	20.0	7–41
Duration of CI-specific clinical Audiology Experience	20 (100%)	19.0	4–40
Experience in Audiology-focused research	12 (60%)	7.5	0–40
Experience in CI-specific research	14 (70%)	12	0–40

**Table 3:** Familiarity ratings for patient-reported outcome measures (PROMs) and clinical outcome measures, ordered by median response. PROMs are shaded in light grey; speech perception tests are unshaded. Numbers under ratings represent the number of participants providing each rating for each measure.

Clinical Measure/PROM	Never Heard	Never Used	Occasionally Used	Regularly Used	Median Response
Speech and Spatial Qualities Scale (SSQ) [41]	0	2	5	13	Regularly Used
Bamford–Kowal–Bench Sentence Test, Australian Version (BKB/A) [42]	0	1	6	10	Regularly Used
City University of New York Sentence Test (CUNY©) [43]	0	2	2	13	Regularly Used
Consonant–Nucleus–Consonant Words (CNC Words) [44]	0	1	0	16	Regularly Used
Arthur Boothroyd Words (AB Words) [45]	0	1	1	15	Regularly Used
Short-Form Speech and Spatial Qualities Scale (SSQ-12) [46]	4	1	1	14	Regularly Used
Hearing Handicap Inventory for the Elderly (HHIE) [47]	2	6	10	2	Occasionally Used
Quick Speech In Noise Test (QuickSIN™) [48]	2	4	9	2	Occasionally Used
Bamford–Kowal–Bench Sentences In Noise Test (BKB-SIN™) [49]	1	5	4	7	Occasionally Used
Digits-In-Noise/Digit Triplet Test (DIN/DTT) [50]	2	3	7	5	Occasionally Used
Glasgow Hearing Aid Benefit Profile (GHABP) [51]	0	9	8	3	Occasionally Used
Abbreviated Profile of Hearing Aid Benefit (APHAB) [52]	2	7	4	7	Occasionally Used
Nijmegen Cochlear Implant Questionnaire (NCIQ) [53]	4	6	9	1	Never Used
Comprehensive Cochlear Implant Questionnaire (CCIQ) [54]	8	5	7	0	Never Used
General Anxiety Disorder-7 (GAD-7) [55]	7	13	0	0	Never Used
Hearing In Noise Test (HINT) [56]	2	8	6	1	Never Used
Revised Hearing Handicap for the Elderly (RHHI) [57]	7	12	1	0	Never Used
Revised Hearing Handicap for the Elderly—Screening (RHHI-S) [57]	8	11	1	0	Never Used
Austin Sentence Test (Austin) [58]	3	6	4	4	Never Used
AzBio Sentence Test (AzBio) [59]	1	12	3	1	Never Used
International Outcomes Inventory—Cochlear Implants (IOI-CI) [60]	2	11	4	3	Never Used
Hearing Aid Users Questionnaire (HAUQ) [61]	9	8	2	1	Never Used
Cochlear Implant Quality of Life Questionnaire—Global (CIQoL-Global) [30]	9	4	7	0	Never Used
Hearing Implant Sound Quality Index (HISQUI19) [62]	9	7	3	1	Never Used
IDA Tool—The Line (The Line) [63]	8	8	2	2	Never Used
Hearing Participation Scale (HPS) [64]	8	11	1	0	Never Used
Hearing Handicap Inventory for the Elderly—Screening (HHIE-S) [65]	4	9	5	2	Never Used
Geriatric Depression Scale—Long (GDS-L) [66]	9	11	0	0	Never Used
Cochlear Implant Quality of Life Questionnaire—Profile (CIQoL-Profile) [30]	9	7	4	0	Never Used
Hearing Device Satisfaction Scale (HDSS) [67]	8	11	1	0	Never Used
Beck’s Depression Index (BDI) [68]	9	10	1	0	Never Used
Depression Anxiety Stress Scale (21 Item) (DASS-21) [69]	8	10	2	0	Never Used
Depression Anxiety Stress Scale (42 Item) (DASS-42) [69]	7	11	2	0	Never Used
Hospital Anxiety and Depression Scale (HADS) [70]	9	5	5	1	Never Used
Bern Benefit in Single-Sided Deafness (BBSS) [71]	10	9	1	0	Never Heard
WHO Wellbeing Index (WHO-S) [72]	10	10	0	0	Never Heard
Expected Consequences of Hearing Aid Ownership (ECHO) [73]	13	7	0	0	Never Heard
Audio Processor Satisfaction Questionnaire (APSQ) [74]	12	7	1	0	Never Heard
De Jong Gierveld Loneliness scale (11 Item) (DJGLS-11) [75]	15	5	0	0	Never Heard
De Jong Gierveld Loneliness scale (6 Item) (DJGLS-6) [76]	15	5	0	0	Never Heard
The Four-Dimensional Symptom Questionnaire (4DSQ) [77]	16	4	0	0	Never Heard
Satisfaction With Life Scale (SWLS) [78]	16	4	0	0	Never Heard
UCLA Loneliness Index (Revised) (UCLA) [79]	16	4	0	0	Never Heard
Visit-Specific Satisfaction Questionnaire (VSQ-9) [80]	19	1	0	0	Never Heard
University of Rhode Island Change Assessment adapted for hearing loss (URICA-HL) [81]	13	7	0	0	Never Heard
Perceived Stress Questionnaire (PSQ) [82]	12	8	0	0	Never Heard
Social Participation Restrictions Questionnaire (SPaRQ) [83]	12	8	0	0	Never Heard
Short Assessment of Patient Satisfaction (SAPS) [84]	14	5	0	1	Never Heard
Satisfaction with Amplification in Daily Life (SADL) [85]	11	9	0	0	Never Heard
Net Promoter Score (NPS) [86]	12	6	2	0	Never Heard
Social Isolation Measure (SIM) [87]	14	6	0	0	Never Heard

**Table 4:** Key findings from the CI user and CI professional final recommendation workshops. * CALD = Culturally and Linguistically Diverse.

	CI Professionals	CI Users
General	*Assessment of all three supra-domains is important.*	*Assessment of all three supra-domains is important.* *Asynchronous remote assessments must consider the amount of time required for the user to complete.* - Completion can be burdensome on the user.
Service Supra-domain	*Preference for simplicity of measurement* - 1-item outcome measures per domain; - Likert/binary outcome scale; - Use of automated methods of data collection to reduce burden of collection and completion.	*Preference for simplicity of measurement* - 1-item outcome measures per domain; - Likert/star rating scale; - Option to expand on answer; - Outcomes should be assessed immediately after service use to ensure responses are contextual. ▪ No more than 3–4 times a year.
	*Technology for remote services should be accessible to everyone* - Consider CALD *; - Important to assess CI users’ ability to use the remote service. ▪ The need to do so is likely to decrease in the future with increased familiarity with technology.	*Technology for remote services should be accessible to everyone* - May depend on end-user connectivity; - CI users should be able to complete remote care sessions independently if required; - Digital literacy is an important consideration.
Clinical Supra-domain	*System checks are essential* - Outcomes are dependent on working hardware.	*System checks are essential* - Should be routine; - Could be performed once a month without the need for patient feedback. ▪ May catch issues quicker than client self-report. ▪ Consider sending a status report to the client.
	*Preference for a minimalist approach focusing on a few key outcome measures* - Recommendation to assess device use and speech perception in noise. ▪ CI outcomes are dependent on CI use.	*Preference for a minimalist approach focusing on a few key outcome measures* - Adaptive tests are often quicker; - Device use. ▪ Important to measure but ultimately up to the CI user to determine how much they wear their device. ▪ Preference for datalogging rather than self-report.
	*Speech perception tests* - Should be suitable for a range of hearing abilities and be “real-world” applicable. ▪ Speech in noise. ▪ Adaptive speech tests. ▪ Need to keep abreast of tests in development, as they may be more appropriate (e.g., ECO-SIN test). - Important to differentiate between diagnostic outcome measures (e.g., confusion matrices to identify which speech sounds are not perceived), which may only be required for some individuals at certain times, and functional outcome measures that assess overall ability to follow speech in quiet and noisy conditions and are often used to track general progress of both individuals and CI groups as a whole.	*Speech perception tests* - Should be “real-world”-applicable.
	*Historical testing* - Recognition that some outcome measures (e.g., speech in quiet) persist for historical reasons. ▪ CI users and CI clinicians to compare outcomes over time. ▪ Use in retrospective outcomes research.	*Historical testing* - Important to be able to compare current and previously measured results to view progress over time.
		*Remote test environment* - Must be considered when implementing speech test outcome measures remotely; - Replication of the same test environment may not be possible. ▪ If this does not matter, it should be communicated to the CI user.
Patient Supra-domain	*PROMs (patient-reported outcome measures)* - Must be practical to implement and use in the clinic. Need to consider: ▪ Length and ease of administration. ▪ Use of a mix of broad and specific PROMs for future COS.	*PROMs* (*patient-reported outcome measures*) - Need to be short and quick to complete. ▪ Maximum 20–25 items. ▪ No mandatory free text items, but there should be free text options. ▪ Should not include multiple items asking similar things. ▪ Must use simple language. - CI users must be made aware that PROMs need to be completed prior to the appointment.
	*Mental Health and Wellbeing* - Can both affect and be affected by hearing loss. - Assessment of this area is vital for the provision of holistic care and support. - May be difficult to distinguish hearing-loss-related mental health issues from those caused by other life stressors. ▪ Hearing-related mental health tools are vital.	*Subjective hearing disability* - PROMs measuring subjective hearing disability are not sensitive enough to pick up deterioration in performance. - Perception that a speech test that reflects “real-life” situations may be more accurate.
	*Satisfaction with CI* - Assessment of satisfaction is crucial because it reflects overall quality of life as well as effectiveness of the CI.	

## Data Availability

The data presented in this study are available on request from the corresponding author due to privacy and ethical restrictions enforced on research relating to human participants by the University of Western Australia.

## Supplementary Materials

The following supporting information can be downloaded at https://www.mdpi.com/article/10.3390/jcm14217697/s1: Figure S1: Bivariate kernel density plots of the correlations between outcome measure ratings; Table S1: PROM description and development information; Table S2: Additional outcome measures using in clinical practice in Australia and New Zealand; Table S3: Factors identified as important to consider when choosing a speech test; File S1: Example survey; File S2: Final Workshop Agenda.

## Abbreviations

CI	Cochlear Implant
CODS	Core Outcome Domain Set
COS	Core Outcome Set
PROM	Patient Reported Outcome Measure
WHO	World Health Organisation
ICF	International classification of Functioning, Disability, and Health framework
COMET	Core Outcome Measures in Effectiveness Trials
