# Supplementary material for: What to Measure? Development of a Core Outcome Set to Assess Remote Technologies for Cochlear Implant Users
Source: J Clin Med. 2025 Oct 30;14(21):7697. doi: 10.3390/jcm14217697 (PMC12609933; doi:10.3390/jcm14217697)
Supplement: Supplementary file 1 [file jcm-14-07697-s001.zip › Supplementary File S1 Example survey.pdf]

Supplementary Data: CI Expert Clinicians Outcomes Survey example

Start of Block: Ethics

The use of remote care telehealth technologies to deliver cochlear implant (CI) services is becoming increasingly available. It is important that both CI service providers, as well as CI researchers, are able to show that the remote care services are at least as good as the "gold-standard" face-to-face clinical model of care. Remote telehealth services can be delivered either as a fully stand-alone telehealth service or as a hybrid model of care that combines both remote telehealth services with in-clinic care.   To compare outcomes between patients, clinics, and research studies, it is essential to use the same set of outcome measures – we call this a Core Outcome Set (COS). While individual patients may have different goals, and therefore different measures of success, these cannot be compared between people, and so it is important that outcome measures included in a COS measure the same underlying constructs for every person. By collecting and analysing the information gained from using the COS, researchers and policymakers can make stronger recommendations about the use and benefits of telehealth services for clinical and research purposes, forming the basis of policy and clinical guidelines. The overall aim of this study is to develop a COS to assess the benefits of remote technologies delivered within adult CI services. This will then allow us to make sure that services can deliver the best care that is personalised for individual CI users. The COS we are looking to develop will include (i) patient outcomes (i.e. benefits of remote technologies for CI patients), and (ii) service delivery, ensuring the outcomes can be easily included in clinical care. This project has been approved by the Human Research Ethics Committee 2022/ET001007.   Please contact Chief Investigator Dr Cathy Sucher: Email catherine.sucher@uwa.edu.au if you have any questions.   Please click on the link below to access the Participant Information Form. Participant Information Form   Thank you for your interest in this research project.

---

*

consent
Please indicate your consent to participate in the survey by responding to the questions below:

- ☐ I have read and understand the information above, and consent to take part in this research project. (1)
- ☐ I understand that I can withdraw at any time, and for any reason, which I do not have to explain, by contacting the research team. (2)

Page Break

This survey is for professionals familiar with the delivery of adult CI services. Please note that we are only reviewing adult cochlear implant services in this study. If this does not apply to you, you may close your browser window to exit, and we thank you for your interest in our research study. Please select which of these best describes you.

○ A hearing professional familiar with the delivery of adult CI services (1)

○ Not a hearing professional familiar with the delivery of adult cochlear implant services (2)

End of Block: Ethics

Start of Block: Demographics

Q47 Section 1

There are seven sections in this survey, each asking different information: information about you, your familiarity with different patient-reported outcome measures (PROMs), your opinion about the PROMs that you have used, your familiarity with different speech perception tests, your opinion about the speech perception tests that you have used, your approach to speech testing in general, a final page thanking you for your time and giving you some more information about the next steps.

Page Break

For how many years have you worked in the following areas? Please click and drag each slider to the appropriate number.

	Not Applicable
	0 10 20 30 40 50
Clinical Audiology ()	
Clinical Cochlear Implant Audiology ()	
Audiology Research ()	
Cochlear Implant Research ()	

In which country do you live?

◯ Australia (1)

◯ New Zealand (2)

Display This Question:

If In which country do you live? = Australia

*

postcode-home-au What is your home postcode?

____

Display This Question:

If In which country do you live? = New Zealand

*

postcode-home-nz What is your home postcode?

____

Display This Question:

If In which country do you live? = Australia

postcode-work-au What is the postcode of the primary location where you work?

____

Display This Question:

If In which country do you live? = New Zealand

postcode-work-nz What is the postcode of the primary location where you work?

____

End of Block: Demographics

Start of Block: PROM Familiarity

Q84 Section 2

Please select how familiar you are with each of the PROMs below. After you select a rating, the next PROM will automatically appear, so you will not have to use the arrows to navigate through unless you wish to change a previous rating.   In the next section, for only those PROMs that you use, or have used, in clinical practice, either regularly or occasionally, we will ask further questions about when they are used and how useful they are, etc. We anticipate that most of your time will be spent answering this next section.

How familiar are you with each of the following patient-reported outcome measures (PROMs) for use in your **clinical practice** (not for research)? There are 41 total items.

	I have never heard of this measure (1)	I have heard of this measure but have never used it in clinical practice (2)	I have used this measure occasionally in clinical practice (3)	I use this measure regularly in clinical practice (4)
Hearing Handicap Inventory for the Elderly (HHIE) (5)	○	○	○	○
Hearing Handicap Inventory for the Elderly - Screening (HHIE-S) (42)	○	○	○	○
Revised Hearing Handicap for the Elderly (RHHI) (15)	○	○	○	○
Revised Hearing Handicap for the Elderly - Screening (RHHI-S) (43)	○	○	○	○
Glasgow Hearing Aid Benefit Profile (GHABP) (6)	○	○	○	○
Abbreviated Profile of Hearing Aid Benefit (APHAB) (7)	○	○	○	○
Bern Benefit in Single-Sided Deafness (BBSS) (8)	○	○	○	○
International Outcomes Inventory - Cochlear Implants (IOI-CI) (9)	○	○	○	○

Nijmegen Cochlear Implant Questionnaire (NCIQ) (10)	○	○	○	○
Hearing Participation Scale (HPS) (11)	○	○	○	○
Comprehensive Cochlear Implant Questionnaire (CCIQ) (12)	○	○	○	○
Cochlear Implant Quality of Life Questionnaire (CIQoL-Global) (13)	○	○	○	○
Cochlear Implant Quality of Life Questionnaire (CIQoL-Profile) (46)	○	○	○	○
Hearing Device Satisfaction Scale (HDSS) (1)	○	○	○	○
Audio Processor Satisfaction Questionnaire (APSQ) (16)	○	○	○	○
WHO Well-being Index (WHO-S) (2)	○	○	○	○
De Jong Gierveld Loneliness scale (11 Item) (45)	○	○	○	○
De Jong Gierveld Loneliness scale (6 Item) (14)	○	○	○	○

The Four Dimensional Symptom Questionnaire (4DSQ) (18)	○	○	○	○
Beck's Depression Index (BDI) (19)	○	○	○	○
UCLA Loneliness Index (Revised) (20)	○	○	○	○
Geriatric Depression Scale - Long (GDS-L) (21)	○	○	○	○
Satisfaction With Life Scale (SWLS) (22)	○	○	○	○
Hospital Anxiety and Depression Scale (HADS) (23)	○	○	○	○
Depression Anxiety Stress Scale (DASS-42) (24)	○	○	○	○
Depression Anxiety Stress Scale (DASS-21) (47)	○	○	○	○
General Anxiety Disorder-7 (GAD-7) (25)	○	○	○	○
Perceived Stress Questionnaire (PSQ) (26)	○	○	○	○
IDA Tool - The Line (27)	○	○	○	○
Hearing Implant Sound Quality Index (HISQUI19) (28)	○	○	○	○

Speech and Spatial Qualities Questionnaire (SSQ) (29)	○	○	○	○
Short Form Speech and Spatial Qualities Questionnaire (SSQ-12) (44)	○	○	○	○
Expected Consequences of Hearing Aid Ownership (ECHO) (31)	○	○	○	○
Hearing Aid Users Questionnaire (HAUQ) (32)	○	○	○	○
Net Promoter Score (NPS) (33)	○	○	○	○
Satisfaction with Amplification in Daily Life (SADL) (34)	○	○	○	○
Short Assessment of Patient Satisfaction (35)	○	○	○	○
Social Participation Restrictions Questionnaire (SPaRQ) (36)	○	○	○	○
Social Isolation Measure (SIM) (37)	○	○	○	○
University of Rhode Island Change Assessment adapted for hearing loss (URICA-HL) (38)	○	○	○	○

Visit-Specific Satisfaction Questionnaire (VSQ-9) (last item in this section) (39)	○	○	○	○

Are there any other questionnaires that you have used in clinical practice with cochlear implant patients?

____

____

____

____

____

End of Block: PROM Familiarity

For any PROMS used regularly or occasionally – the following questions were posed

Q90 Section 3
Note that in this section you will only see those PROMs that you previously indicated you have used.

Do you use (or have you used) this PROM ***(i.e. any marked as previously used)*** with patients using cochlear implants and/or both hearing aids and cochlear implants (i.e. bimodal users)?

- ☐ Cochlear Implants (2)
- ☐ Both Hearing Aids and Cochlear Implants (Bimodal) (5)

With your clinical patients, how do you usually use this PROM? Click all that apply.

- ☐ Before a rehabilitation program to assist with planning (1)
- ☐ During a rehabilitation program to assess progress (2)
- ☐ After a rehabilitation program to assess outcomes (3)
- ☐ Other: (4) ____

How much do you agree with the following statements?

	Strongly disagree (-2)	Somewhat disagree (-1)	Neither agree nor disagree (0)	Somewhat agree (1)	Strongly agree (2)	Don't know (-99)
This PROM gives results that are useful in clinical practice (1)	○	○	○	○	○	○
This PROM gives results that are trustworthy/believable (2)	○	○	○	○	○	○
This PROM is easy to use in clinical practice (3)	○	○	○	○	○	○
I would use this PROM in clinical practice if it were recommended to me (4)	○	○	○	○	○	○

Do you have any comments regarding this PROM? (optional)

____

____

____

____

____

Start of Block: Speech Familiarity

Section 4

In this section, you will be asked about clinical measures commonly used in assessing sound clarity and acceptability.   Please select how familiar you are with each of the **clinical measures** below. After you select a rating, the next clinical measure will automatically appear, so you will not have to use the arrows to navigate through unless you wish to change a previous rating.   In the next section, for only those clinical measures that you use, or have used, in clinical practice, either regularly or occasionally, we will ask further questions about when they are used and how useful they are, etc. We anticipate that most of your time will be spent answering this next section.

familiarity-speech How familiar are you with each of the following clinical measures for use in your **clinical practice** (not for research)? There are 10 total items.

	I have never heard of this measure (1)	I have heard of this measure but have never used it in clinical practice (2)	I have used this measure occasionally in clinical practice (3)	I use this measure regularly in clinical practice (4)
Arthur Boothroyd Words (AB Words) (5)	○	○	○	○
Consonant-Nucleus-Consonant Words (CNC Words) (42)	○	○	○	○
City University of New York Sentence Test (CUNY Sentences©) (15)	○	○	○	○
Bamford-Kowal-Bench Sentence Test, Australian Version (BKB/A) (38)	○	○	○	○
AzBio Sentence Test (49)	○	○	○	○
Austin Sentence Test (43)	○	○	○	○
Bamford-Kowal-Bench Sentences In Noise Test (BKB-SIN™) (50)	○	○	○	○
Quick Speech In Noise Test (QuickSIN™) (51)	○	○	○	○
Hearing In Noise Test (HINT) (6)	○	○	○	○

Digits-In-Noise/Digit Triplet Test (DIN/DTT) (last item in this section) (52)	○	○	○	○

If there are any other **speech perception** tests that you use as standard, please list them below.

____

If there are any other **music perception** tests that you use as standard, please list them below.

____

If there are any other **environmental sound perception** tests that you use as standard, please list them below.

____

If there are any other **electrophysiological** tests (e.g. eABR, Speech corticals, etc.) that you use as standard, please list them below.

____

End of Block: Speech Familiarity

Section 5

Note that in this section you will only see those clinical measures that you previously indicated you have used (regularly or occasionally).

Why do you use this measure? (indicate all options that are appropriate)

- ☐ It is part of our standard clinical protocol pre-operatively (1)
- ☐ It is part of our standard clinical protocol post-operatively (2)
- ☐ The assessment materials are readily available for me in the clinic (3)
- ☐ The test difficulty is suitable for the majority of our clients (limited ceiling and floor effects) (4)
- ☐ The test gives a good indication of how my client is managing in "real life" (5)
- ☐ It is what is most commonly reported on in the literature (6)
- ☐ To compare the results with my client's previous results (7)
- ☐ To compare the results with the results of groups of clients (own clinic) (8)
- ☐ To compare the results with the results of groups of clients (other clinics) (9)
- ☐ Other - please state: (10) ____________________

For the majority of my clients, I present this test in the following manner: (mark all options that are appropriate; please ensure that either at least one option is selected within each group, or that you have filled in the "other" section at the bottom)

- ☐ Live (1)
- ☐ Recorded (2)
- ☐ Auditory Alone (3)
- ☐ Auditory Visual (5)
- ☐ Visual Alone (6)
- ☐ Freefield stimulus from the front (7)
- ☐ Freefield stimulus spatially separated (8)
- ☐ Direct audio input (9)
- ☐ Audio streaming (10)
- ☐ Under headphones (11)
- ☐ In quiet (12)
- ☐ In adaptive noise (13)
- ☐ In fixed noise +10dB S:N (14)
- ☐ In fixed noise +5dB S:N (15)
- ☐ In fixed noise 0dB S:N (16)
- ☐ Another noise condition - please state: (21)

____

☐ Each ear separately (17)

☐ Binaural (18)

☐ Other - please state: (19) ____

What presentation level do you most commonly use to present the test material (e.g. 60 dBHL, 40 dBSPL, 70 dBA)?

____

How much do you agree with the following statements?

	Strongly disagree (-2)	Somewhat disagree (-1)	Neither agree nor disagree (0)	Somewhat agree (1)	Strongly agree (2)	Don't know (-99)
This test gives results that are useful in clinical practice (1)	○	○	○	○	○	○
This test gives results that are trustworthy/believable (2)	○	○	○	○	○	○
This test is easy to use in clinical practice (3)	○	○	○	○	○	○
I would use this test in clinical practice if it were recommended to me (4)	○	○	○	○	○	○

Section 6

How do you usually test **asymmetrical hearing losses**? (mark all options that are appropriate)

- [ ] Masking the better ear (please indicate masking level in dB) (1) ____
- [ ] Blocking or plugging the better ear (2)
- [ ] Using an ear muff over the better ear (3)
- [ ] Using DAI (4)
- [ ] Using audio streaming (5)
- [ ] Other - please state: (6) ____

Are there any other factors that are important to you when considering use of a speech perception test? (e.g. presentation language, accent, etc.)

____

Section 7
Thank you for completing the survey.

Please leave any comments about the survey here.

____

Please indicate if you wish to receive the results of this research on completion of the study.

- [ ] Yes (1)

Please enter your email address:

____
