# Supplementary material for: What to Measure? Development of a Core Outcome Set to Assess Remote Technologies for Cochlear Implant Users
Source: J Clin Med. 2025 Oct 30;14(21):7697. doi: 10.3390/jcm14217697 (PMC12609933; doi:10.3390/jcm14217697)
Supplement: Supplementary file 1 [file jcm-14-07697-s001.zip › Supplementary File S2 Final Workshop Agenda.pdf]

Final Workshop: Development of a Core Outcome Set to evaluate remote technologies for cochlear implant users

Agenda

Attendees: (TBA)

Workshop Leaders: Melanie Ferguson, Cathy Sucher, Isabelle Boisvert

1. Short presentation on outcomes from previous workshops and Delphi reviews

2. Workshop Questions
    a. If you have utilised remote assessments (either as a professional or as a CI recipient), what has your experience been when using them?
    b. What are your thoughts/feelings about the key outcome measures identified in the earlier parts of the study in relation to their use for remote management and assessment of CI users?
        i. Benefits in general and compared to current clinical measures
        ii. Limitations in general and compared to current clinical measures
    c. Do you have any suggestions for outcome measures for the key domains that we have not yet identified?
    d. What do you think are the best modalities (e.g. surveys, DAI, streaming etc) to use when assessing outcomes for remote technologies for CI?
    e. Do you feel that we have missed any important points regarding the outcome measures we have covered, or other outcome measures we have overlooked?
    f. Is there any additional information, access, or support you require in order to to consider using, or continue using remote assessment to access your own, or our client's CI outcomes?
