# Supplementary material for: What to Measure? Development of a Core Outcome Set to Assess Remote Technologies for Cochlear Implant Users
Source: J Clin Med. 2025 Oct 30;14(21):7697. doi: 10.3390/jcm14217697 (PMC12609933; doi:10.3390/jcm14217697)
Supplement: Supplementary file 1 [file jcm-14-07697-s001.zip › Supplementary Table S2.pdf]

Supplementary Table S2: Additional outcome measures used in clinical practice in Australia and New Zealand not presented in the initial survey.

Type of Tool	Outcome measured	Name of Tool	Respondents suggesting tool (n)
Patient Reported Outcome Measures (PROMs)	Goal-based outcomes	Client Oriented Scale of Improvement (COSI)	1
		Outcome Rating Scale (ORS)	2
	Tinnitus outcomes	Tinnitus Reaction Questionnaire (TRQ)	2
		Tinnitus Handicap Inventory (THI)	2
		Iowa Tinnitus Handicap Questionnaire	1
		Tinnitus Functional Index (TFI)	2
	General measures of (Health-related) Quality of Life	Health Utilities Index Mark 3 (HUI-3)	1
		Australian Quality of Life Scale (AQoL)	4
	psychological and cognitive state and functioning	General Behavior Inventory (GBI)	1
		Strengths and Difficulties Questionnaire (SDQ)	3
		Mini Mental State Exam (Mini-Cog©)	2
		Hospital Anxiety and Depression Scale	1
	general hearing rehabilitation	Hearing Handicap Inventory for Adults (HHIA)	2
		Hearing Handicap Inventory for Adults – Screening (HHIA-S)	1
	Third-Party Disability	Significant Other Scale for Hearing Disability (SOS-HEAR)	1
	Listening Effort	Listening Effort Questionnaire – Cochlear Implants (LEQ-CI)	3
	Assessments for states that may impact on rehabilitation	Groningen Frailty Index (GFI)	1
		Weschler Individual Achievement Test Second Edition (WIAT-II)	1
	Cochlear Implant-specific measures	Categories of Auditory Performance (CAP-9)	1
		Internally developed questionnaires	4
	Music Perception	Munich Music Questionnaire	1
	Environmental sound perception	Environmental Sounds Checklist of the NAL Listening Checklist	1
Clinical	Speech sound perception	Ling Sounds	3
		Modified Glendonald Auditory Screening Procedure (GASP)	1
		Iowa Medial Consonant Test	1
		Everyday Conversational Sentences in Noise (ECO-SiN)	1
		Test of Auditory Processing Skills (TAPS-4)	1
		Monosyllable, Spondee, Trochee, Polysyllable (MSTP) tests (e.g. PLOTT)	1

	Narrative assessment of comprehension	1
Music Perception	Spectro-Temporal Ripple for Investigating Processor Effectiveness (STRIPES/webSTRIPES	
Electrophysiological measures	Electrocortical measures	6
	Electrically-Evoked Stapedial Reflex Threshold (eSRT)	2
	Neural Response Telemetry (NRT) / Auditory Response Telemetry (ART)	3
	Electrically-evoked Acoustic Brainstem Response	1
