# Supplementary material for: What to Measure? Development of a Core Outcome Set to Assess Remote Technologies for Cochlear Implant Users
Source: J Clin Med. 2025 Oct 30;14(21):7697. doi: 10.3390/jcm14217697 (PMC12609933; doi:10.3390/jcm14217697)
Supplement: Supplementary file 1 [file jcm-14-07697-s001.zip › Supplementary Table S3.pdf]

Supplementary Table S3: Factors identified as important to consider when choosing a speech test.

	Factor	Number of respondents (n)
**Patient Specific Factors**	Primary language(s) of the patient relative to the available language(s) of the test	11
	Accent used in test materials	7
	Speed of delivery	2
	Length of test	2
	Appropriateness of the test to the patient's cognitive ability/available cognitive reserve	4
**Test Specific Factors**	Availability of automated scoring	1
	Contemporaneity of test materials	1
	Room acoustics	1
	Presence of ceiling and floor effects	1
	Test flexibility	1
