# Supplementary figures and images for: What to Measure? Development of a Core Outcome Set to Assess Remote Technologies for Cochlear Implant Users
Source: J Clin Med. 2025 Oct 30;14(21):7697. doi: 10.3390/jcm14217697 (PMC12609933; doi:10.3390/jcm14217697)

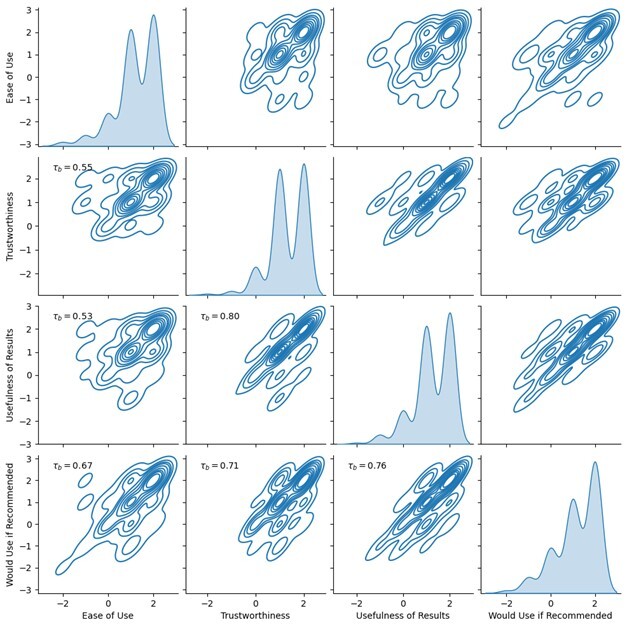

Supplement: Supplementary file 1 [file jcm-14-07697-s001.zip › Supplementary Figure S1.jpg]
